# An Ethnobotanical Investigation into the Traditional Uses of Mediterranean Medicinal and Aromatic Plants: The Case of Troodos Mountains in Cyprus

**DOI:** 10.3390/plants12051119

**Published:** 2023-03-02

**Authors:** Andreas M. Savvides, Constantina Stavridou, Sotiroula Ioannidou, Christos Zoumides, Andreas Stylianou

**Affiliations:** 1 Agricultural Research Institute, P.O. Box 22016, Nicosia 1516, Cyprus; 2 Energy, Environment and Water Research Center (EEWRC), The Cyprus Institute, P.O. Box 27456, Nicosia 1645, Cyprus

**Keywords:** ethnobotany, cultural importance index, cultural value, traditional knowledge, medicinal uses, culinary uses

## Abstract

The Troodos mountains in Cyprus are a hotspot of plant diversity and cultural heritage. However, the traditional uses of medicinal and aromatic plants (MAPs), a significant aspect of local culture, have not been thoroughly studied. The aim of this research was to document and analyze the traditional uses of MAPs in Troodos. Data on MAPs and their traditional uses were collected through interviews. A database was constructed with categorized information on the uses of 160 taxa belonging to 63 families. The quantitative analysis included the calculation and comparison of six indices of ethnobotanical importance. The cultural value index was selected to reveal the most culturally significant MAPs taxa, while the informant consensus index was utilized to quantify the consensus in information obtained related to uses of MAPs. Furthermore, the 30 most popular MAPs taxa, exceptional and fading uses, and the plant parts used for different purposes are described and reported. The results reveal a deep connection between the people of Troodos and the plants of the area. Overall, the study provides the first ethnobotanical assessment for the Troodos mountains in Cyprus, contributing to a better understanding of the diverse uses of MAPs in mountain regions of the Mediterranean.

## 1. Introduction

Ethnobotany is the field of study that examines the complex interrelations between humans and plants [1], with its roots dating back to ancient times, and references found in historical texts, such as the Old Testament of the Bible [2]. Traditional ethnobotanical knowledge safeguards eroding folk traditions and contributes to the preservation of cultural heritage [3,4]. Furthermore, ethnobotany plays a significant role in the discovery and rediscovery of medicines derived from plants [5] and phytonutrients that provide numerous health benefits [6]. Additionally, the knowledge obtained contributes to both biological and cultural diversity [7,8]. The coexistence of indigenous cultures with their environment is governed by a symbiotic and balanced relationship, where the recording of traditions and their accumulated knowledge of the biodynamics of the natural world, the management of land reserves and plant communities, help to maintain the sustainability of the ecosystem [9]. However, the declining use of traditional ecological knowledge, especially in small Mediterranean island communities, threatens their role as biocultural refugia (i.e., places that shelter species and people who are transmitters of traditional ecological knowledge and practices) and their potential to be genetic and cultural pools for a vast range of species co-evolving with humans for centuries [10,11].

Medicinal plants are primarily used in traditional and modern medicine, while aromatic plants are mainly utilized for their aroma and flavor. However, many plants defined as medicinal and aromatic plants (MAPs) are also used for various other purposes, such as cosmetics, dyes—coloring agents, and plant protection products, as well as in religious observances [12], thus increasing the diversity of their uses. The activities of early humans in relation to MAPs are still maintained today, with the exploration of plants and botanical sourcing for new uses [13]. Due to their beneficial active ingredients, MAPs present an expanded commercial potential, as they are used as a source of raw material both by the pharmaceutical industry and in the production of home remedies and herbal products [14]. The rates of global use, demand, and trade in MAPs products have increased significantly over time, indicating, on the one hand, the economic importance of these commodities, and, on the other hand, the need for promoting the conservation and sustainable uses of MAPs [15].

**Figure 1:**
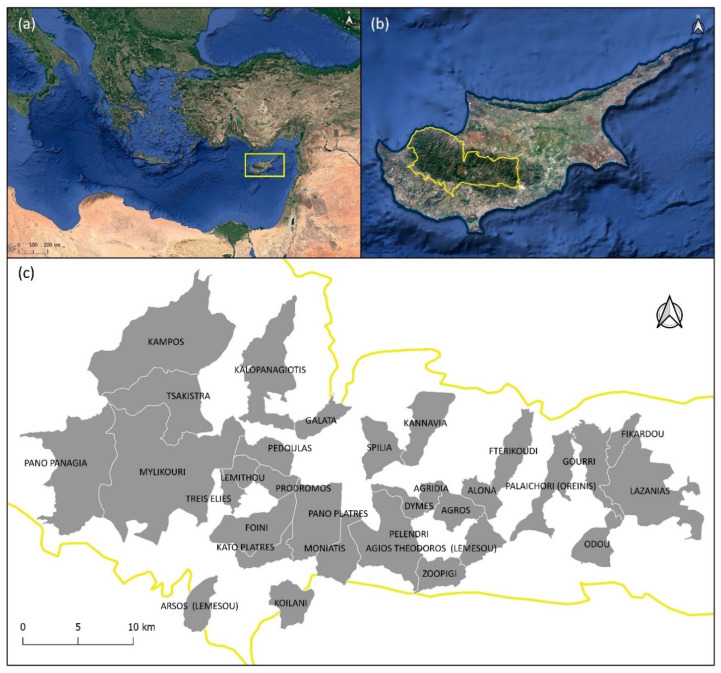
(**a**) The island of Cyprus (yellow rectangle; ©2022 Google). (**b**) Troodos Mountain Range (©2022 Google) covers the second phytogeographical division of the island (yellow line, [22]). (**c**) The 31 communities (grey shapes separated by white lines) included in the study area (yellow line).

The Mediterranean basin is known as a global hotspot for both endemic vascular plants and the traditional knowledge related to the use of MAPs [3,16]. Cyprus also exhibits these characteristics, especially in the mountainous areas of Troodos. Cyprus, the third largest island in the Mediterranean Sea, is situated in the eastern Mediterranean, at the crossroads of the Middle East, North Africa, and Europe (Figure 1a). Throughout history, it has been an important center for commerce, such as in herbal trade [17], and the meeting point of multiple civilizations [18]. The commercial relations between Cyprus and neighboring countries are evident, particularly in the exchange of goods derived from MAPs, such as oils, perfumes, and spices [19]. The island has diverse landscapes and supports high species and habitat diversity [20]. The diverse flora of the island comprises of approximately 2000 native taxa [21,22,23]. Many of these taxa are considered MAPs and are mentioned by writers from antiquity [24], to medieval [25], and modern times [19], with emphasis on their medicinal and aromatic properties.

Cyprus is home to two mountain ranges, namely Troodos and Pentadaktylos. Troodos, the largest and highest mountain range (altitude 1952 m) of the island, covers most of its western part (Figure 1b) and hosts a high number of MAPs due to its climatic and geomorphological conditions. Troodos receives the highest precipitation and the lowest temperatures on the island and is characterized by a Supra-Mediterranean-Humid bioclimate [20]. The mountain range comprises an ophiolite complex with dominant basic and ultrabasic rocks and is surrounded by sedimentary formations [20], adding to the geo-climatic distinctiveness of the area.

To illustrate the distribution of plant species in Cyprus, Meikle [22] divided the island into eight phytogeographical divisions. Troodos range (i.e., second phytogeographical division; Figure 1b), with 1127 taxa (species and subspecies), of which 102 are endemic (72% of the island) [23], is a hotspot for plant diversity. In addition to its natural vegetation, Troodos is known for its vineyards, which have a long history in Cyprus [26]. Vineyards are cultivated in semi-mountainous and mountainous areas [27] as well as terraced orchards of fruit and nut trees, which extensively feature in the area due to their climatic requirements [27,28,29]. The oldest evidence of agricultural land use in Troodos dates back to prehistoric times [30]. The long agrarian history among the inhabitants, coupled with their physical isolation within a natural heritage site, such as the Troodos Mountain Range, suggests a traditionally strong and remarkable relationship between people and MAPs. However, urbanization and depopulation [27] threaten the preservation and transfer of the current knowledge and understanding of the traditional uses of MAPs in the Troodos region.

The contribution of MAPs in scientific fields, such as the agricultural and environmental sciences, in Cyprus has been documented by studies in recent years [31,32,33,34], indicating the growing interest of the scientific community in their potential and exploitation. Despite the high interest in MAPs and their diverse uses, the available information on traditional MAPs uses in Cyprus is scarce and scattered and focuses on the island as a whole. A typical example is the handwritten “Iatrosophikon” collection of Mitrophanes (1790–1867), a custodian and monk at the Mahairas Monastery, which contains recipes for treating various diseases, primarily using MAPs [25]. Efforts have been made to preserve this information, such as the list of medicinal plants of Cyprus [35], a description of the therapeutic properties and recipes for use of 673 herbs [36], a record of wild edible plants in Larnaka and Pafos provinces [37], as well as a systematic documentation of MAPs in Cyprus from antiquity to the present day [19].

Considering the absence of an ethnobotanical study on the traditional uses of MAPs specifically for the Troodos Mountain region, the aims of the present study were to (1) collect and document information about the traditional and diverse uses of MAPs by local people in Troodos mountainous communities, (2) calculate and compare different indices of ethnobotanical importance, and (3) determine the most culturally significant taxa based on the calculated indices.

## 2. Results and Discussion

### 2.1. Demographics

The main demographic characteristics (gender, age, and education) of the respondents in the ethnobotanical study are presented in Table 1. Most respondents were women (54%), which is consistent with previous research in Greece (60%) [38], but non-consistent with studies in Spain (40%) [39,40] and Italy (10%) [41]. This finding indicates the important and active role of women in Troodos, both in the use and in the transmission of knowledge about MAPs. Several oral reports also suggest that in the past, many women were engaged in practices that involved the use of MAPs. Nevertheless, the role of men should not be underestimated, particularly in the transmission of knowledge, as demonstrated by the relatively high percentage (46%) of male respondents.

The age of the respondents is of particular interest, with an average age of 73 years old and a majority of 54% falling within the 70–89 age group, followed by the 50–69 age group (28%). Compared to a similar study in Cyprus (average age 63 years) and other Mediterranean countries (e.g., Italy 63, Spain 65, Algeria 65, Morocco 55, Egypt 50 [9]), the average age in the present study confirms the aging of knowledge transmitters.

Moreover, most participants (67%) had completed at least secondary education. These findings suggest that the retention and continuation of knowledge is primarily dependent on older individuals and those with relatively high educational level. Additionally, participants frequently referred to deceased individuals who possessed more knowledge, both about plant taxa and their uses, emphasizing the considerable loss of valuable knowledge and the imperative need for its preservation.

### 2.2. Use Categories and Sub-Categories

**Figure 2:**
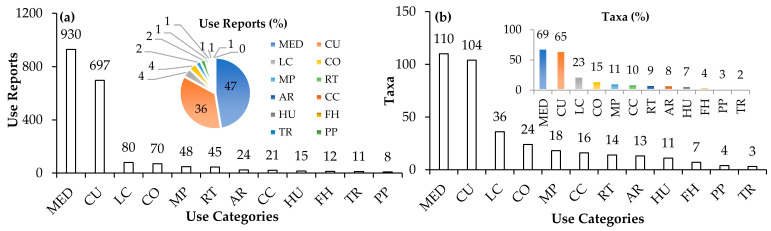
(**a**) The number of use reports (bar chart) and their percentage (pie chart) by use category. (**b**) The number of taxa reported (bar chart) and their percentage of the total number of taxa reported in the ethnobotanical study (colored bar chart) by use category. Use categories: medicinal (MED), culinary (CU), livestock care (LC), cosmetics (CO), manufacture–processing (MP), religious traditions (RT), animal repellent (AR), crop care (CC), household use (HU), fishing–hunting (FH), trade (TR), poisonous plants (PP).

The most common use category reported was the medicinal (MED) category, constituting 47% of the total use reports (URs) as depicted in Figure 2a, followed by the culinary (CU) category with 36%. The remaining use categories accounted for 17% in total, with reported taxa per use category being less than 10%. Regarding the number of reported taxa (Figure 2b), the MED category had the highest diversity (69%), followed by CU (65%), livestock care (LC;23%), cosmetics (CO;15%), manufacture-processing (MP;11%), and crop care (CC;10%). The reported taxa per use category for the remaining use categories were less than 10%.

**Figure 3:**
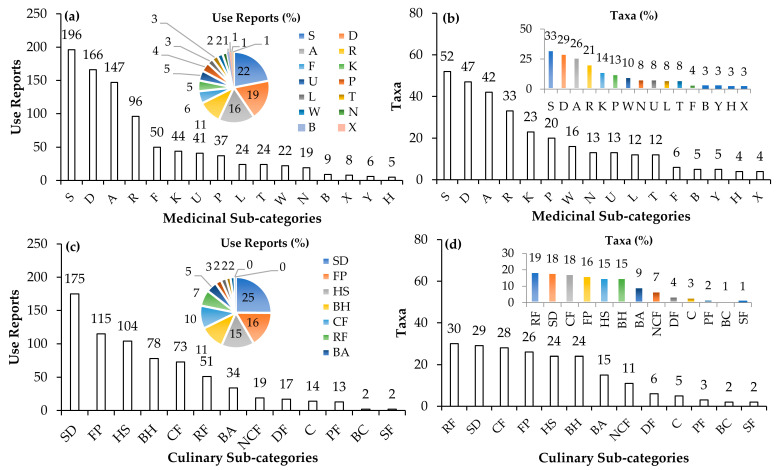
(**a**) The number of use reports (bar chart) and their percentage (pie chart) by medicinal sub-category. (**b**) The number of plant taxa reported (bar chart) and their percentage (colored bar chart) by medicinal sub-category. (**c**) The number of use reports (bar chart) and their percentage (pie chart) by culinary sub-category. (**d**) The number of plant taxa reported (bar chart) and their percentage (colored bar chart) by culinary sub-category. Medicinal sub-categories (according to ICPC-2): skin (S), digestive (D), general and unspecified (A), respiratory (R), eye (F), cardiovascular (K), urological (U), psychological (P), musculoskeletal (L), endocrine/metabolic and nutritional (T), pregnancy, childbearing (W), neurological (N), blood, blood forming organs and immune mechanism (B), female genital (X), male genital (Y), ear (H). Culinary sub-categories: sweets and desserts (SD), food preservation (FP), herbs & spices, (HS), hot beverage (tea; BH), cooked food ingredient (CF), raw food (RF), alcoholic beverage (BA), non-cooked food ingredient (NCF), dried fruits (DF), condiment (other; C), pickled food (PF), cold beverage (BC), smoke flavoring (SF).

Based on the number of URs (Figure 3a), the most important MED sub-category was for skin issues (22%), followed by digestive (19%), general and unspecified (16%) and respiratory issues (11%). The remaining sub-categories gathered less than 10% each and accounted for 32% of the total URs. When looking at the number of taxa reported for the MED sub-categories (Figure 3b), the highest diversity was for skin issues (33%), followed by digestive (29%), general and unspecified (26%), respiratory (21%), cardiovascular (14%), psychological (13%) and pregnancy–childbearing (10%). The remaining MED sub-categories accounted for less than 10% of the reported taxa. It is worth noting that the general and unspecified sub-category was highly cited due to the general issues included such as chills/fever, general pain, and weakness/tiredness; issues that may result out of several unspecified factors.

Regarding the CU sub-categories (Figure 3c), sweets and deserts were the most frequently reported sub-category (25%), followed by food preservation (16%), herbs and spices (15%), hot beverage (11%), and cooked food ingredients (10%). The remaining sub-categories (23% in total) accounted for less than 10%. In terms of the number of taxa reported (Figure 3d), raw food had the highest diversity (19%), followed by sweets and desserts (18%), cooked food ingredient (18%), food preservation (16%), herbs and spices (15%), and hot beverage (15%). The remaining reported taxa per CU sub-category accounted for less than 10% each.

Most of the use categories (Figure S1a), MED sub-categories (Figure S1b), and CU sub-categories (Figure S1c) had an Informant Consensus Factor (ICF) higher than 0.50, indicating high levels of consensus in information at these levels. The highest ICF was found in MED (ICF = 0.88), CU (ICF = 0.85), and trade (ICF = 0.80), while the lowest ICF was in household use (ICF = 0.29) and crop care (ICF = 0.25), indicating disagreement among respondents regarding the taxa used for these uses. Among the MED sub-categories, eye (ICF = 0.90), skin (ICF = 0.74), digestive (ICF = 0.72), general and unspecified (ICF = 0.72), and urological issues (ICF = 0.70) showed the highest ICF, while neurological (ICF = 0.33), pregnancy, childbearing (ICF = 0.29), ear (ICF = 0.25), and male genital issues (ICF = 0.20) showed the lowest ICF. Among the CU sub-categories, sweets and desserts (ICF = 0.84), pickled food (ICF = 0.83), food preservation (ICF = 0.78), and herbs and spices (ICF = 0.78) showed the highest ICF, while cold beverage (ICF = 0), and smoke flavoring (ICF = 0) showed the lowest ICF.

### 2.3. Plant Taxa Characterization

The 160 plant taxa mentioned in the interviews and identified as MAPs belong to two divisions: Spermatophyta (n = 157) and Pteridophyta (n = 3). The taxa are distributed across 63 different families; 144 are separate species or subspecies/varieties and 16 are genera. Fifty-four percent of these taxa were exclusively collected from the wild, 36% were exclusively cultivated, 5% were either collected or cultivated, 1% were either imported or cultivated, and 4% were plants whose products were imported. Of the 144 species, 69 are indigenous, with eight being endemic and one near-endemic to Cyprus. The majority of the 16 recorded genera are native plants, with some being endemic. A notable proportion of the traditionally used plants are not indigenous and are cultivated.

**Figure 4:**
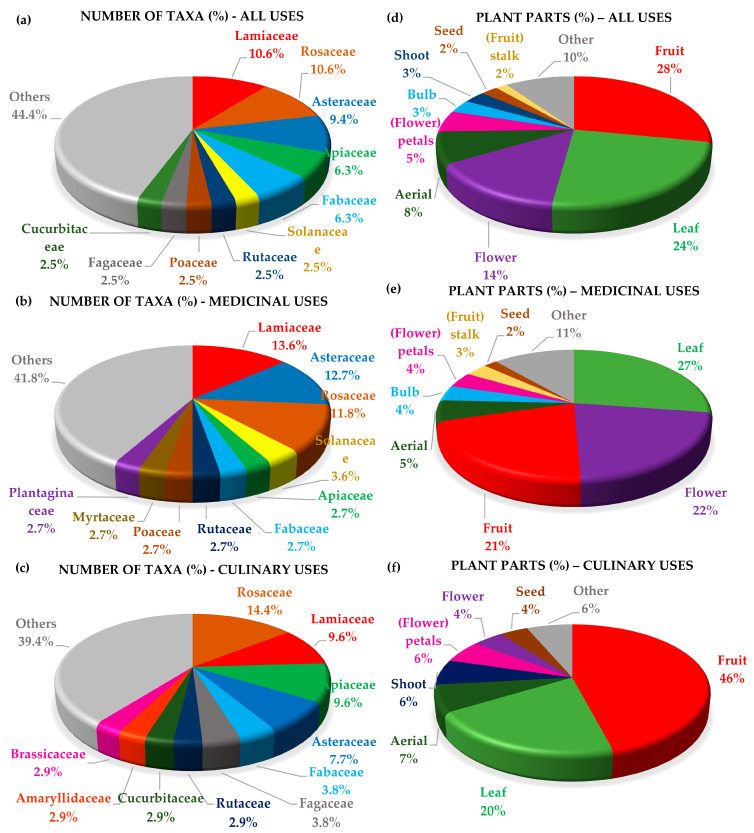
The percentage of reported taxa per plant family across all uses (**a**), with respect to medicinal uses (**b**), and culinary uses (**c**). The percentage of reported used plant parts across all uses (**d**), with respect to medicinal (**e**) and culinary uses (**f**).

In terms of all use categories, most recorded taxa belong to the families Lamiaceae (11%), Rosaceae (11%), Asteraceae (9%), Apiaceae (6%), and Fabaceae (6%) (Figure 4a). As regards MED uses specifically, most taxa belong to the families Lamiaceae (14%), Asteraceae (13%), and Rosaceae (12%) (Figure 4b).

The most used families for CU uses are Rosaceae (14%), Lamiaceae (10%), Apiaceae (10%), and Asteraceae (8%) (Figure 4c). These findings are consistent with previous studies of the herbal market in Cyprus [17] and historical texts and Greek Orthodox monasteries in Cyprus [42], especially in the context of MED uses.

In terms of all uses (Figure 4d), the most recorded plant parts used were the fruit (28%), the leaf (24%), the whole flower (14%) and flower petals (5%) followed by the aerial part of the plant (8%), the bulb (3%), the shoot (3%), and the seed (2%). Other plant parts accounted for 10% of all the reported plant parts. Regarding MED uses (Figure 4e), the most recorded plant parts used were the leaf (27%), the flower (22%) and flower petals (4%), and the fruit (21%) followed by the aerial part of the plant (5%), the bulb (4%), the fruit stalk (3%), and the seed (2%). Other plant parts accounted for 11% of all the reported plant parts. Finally, in terms of CU uses (Figure 4f), the most recorded plant parts used were the fruit (46%), and the leaf (20%) followed by the aerial part (7%), the shoot (6%), the flower petals (6%) and whole flower (4%), and the seed (4%). Other plant parts accounted for 6% of all the reported plant parts.

Table S1 organizes the 160 plant taxa recorded in the ethnobotanical study, classified by plant division and family. It provides the scientific and vernacular names of the taxonomic units, as well as information on their indigenous or endemic status, collection/cultivation/importation status (their products) and use categories. Additionally, Table S1 includes the MED and CU uses sub-categories and their ranking based on the cultural index values for all uses.

### 2.4. Comparison of Different Ethnobotanical Importance Indices

Different ethnobotanical indices have been used throughout the years to identify the most culturally significant or popular taxa in several studies [43,44,45]. In this study, six different indices were calculated, based on the data obtained and were compared with each other using Spearman’s correlation coefficient. We selected an index that incorporates significant variables and showed strong correlations with some of the most widely used ethnobotanical indices.

**Figure 5:**
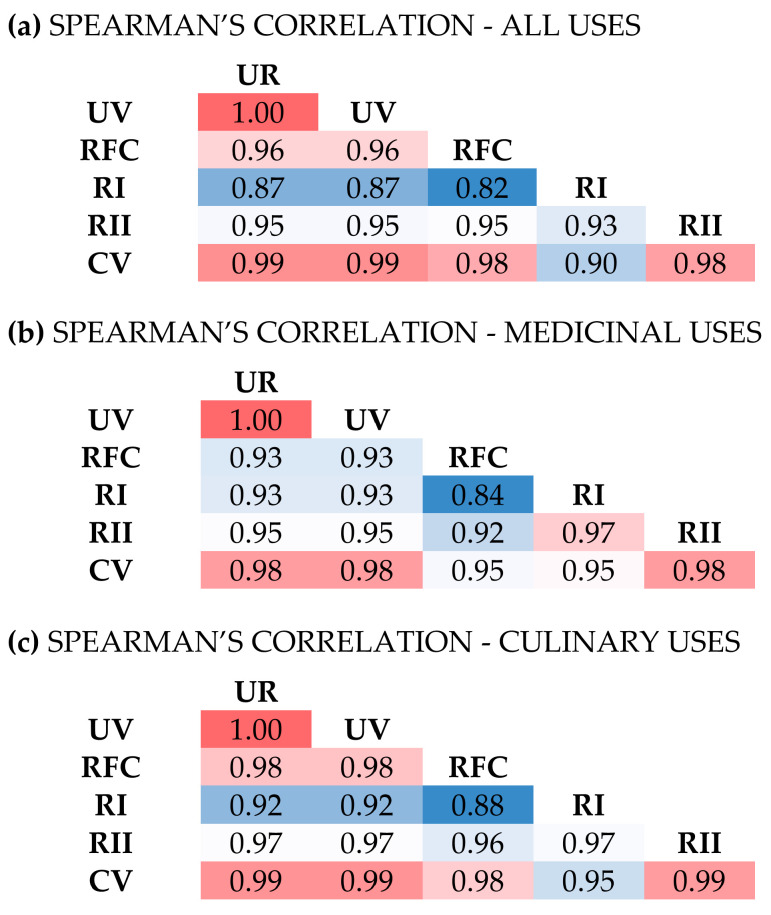
Spearman’s correlation matrices indicating the correlations among the number of use reports (UR), the use value (UV), the relative frequency of citation (RFC), the relative importance (RI), the relative importance index (RII), and the cultural value index (CV) for all uses (**a**), medicinal uses (**b**), and culinary uses (**c**). Spearman’s coefficient, the value in each cell for each combination of variables, is a measure of the strength and direction (negative or positive) of the association between the two indices and ranges from 0 (negligible association) to 1 (very high association). The lowest values per matrix are noted with dark blue color while the highest with dark red color, low and high values are noted using lighter blue and red colors, respectively, while intermediate values are white.

The study found that the six different indices used were highly correlated with each other, and the pattern of correlation remained consistent when the indices were calculated based on all uses (Figure 5a), MED uses only (Figure 5b), or CU uses only (Figure 5c). This finding coincides with the results of a previous study by Coe and Gaoue [46], which suggests that most cultural importance indices do not measure unique aspects, but rather they tend to overlap in what they aim to quantify. In another comparative study, high associations were also observed among different indices, concluding that the more versatile a plant regarding its uses the more widespread its usefulness [45]. The most widely used indices used in the current study are the Use Reports (UR), the number of reports per taxon studied and the Use Value (UV) [43]. UR and UV are expected to be highly associated across taxa, as UV equals UR divided by the total number of participants; groups (Figure 5). UR and UV were well associated with the Relative Frequency of Citation (RFC), an index accounting for the number of informants’ groups mentioning the selected species, indicating that the number of use reports is well associated with the number of informants’ groups for a selected species. The lowest Spearman’s coefficient was observed between RFC and Relative Importance (RI); the latter being a measure of the versatility of the uses that does not account for the number of informants or the use reports. In general, RI showed the lowest associations with other indices (Figure 5). The Relative Importance Index (RII) depends on the frequency of citation and the versality of the uses of a selected taxon in relation to their maximum values observed in the study. RII, UR, UV, RFC, and at a lesser degree RI, showed high associations with the Cultural Value Index (CV), an index considering the number of use categories, the relative frequency of citation and the number of use reports of a selected taxon (Figure 5). The research team ultimately determined that CV was the best index to use for understanding taxa popularity and cultural importance in traditional uses of MAPs in the mountainous areas of Troodos, as it was not only strongly correlated with other important ethnobotanical indices, but also takes into account the versality of the uses, the frequency of citation, and the number of use reports.

### 2.5. Culturally Significant Plant Taxa Used in Troodos Mountains

**Figure 6:**
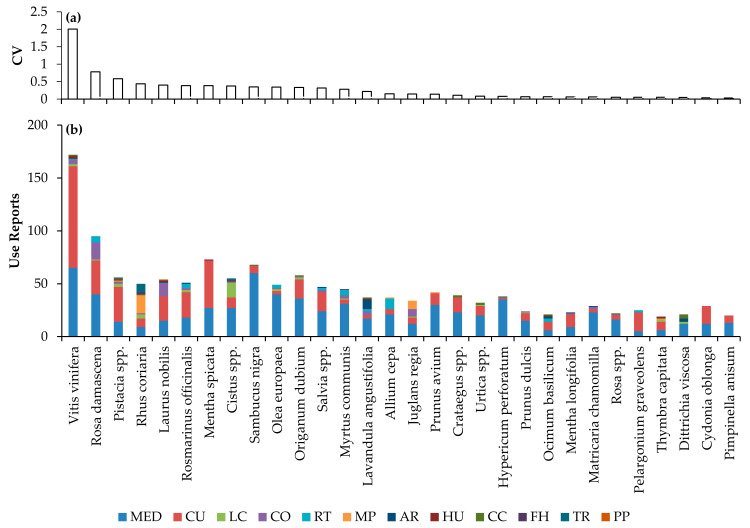
(**a**) The cultural value (CV), considering all uses, of the 30 more culturally significant taxa. (**b**) The number of use reports per taxa (bars) per use category (different colors per bar). Use categories: medicinal (MED), culinary (CU), livestock care (LC), cosmetics (CO), manufacture–processing (MP), religious traditions (RT), animal repellent (AR), crop care (CC), household use (HU), fishing–hunting (FH), trade (TR), poisonous plants (PP).

The people of Troodos have a long-standing tradition of using a unique blend of mainly locally sourced plants for their various needs (Table S1), as specifically evidenced by the 30 most significant (based on CV index) taxa identified in this study, considering all uses (Figure 6a). Figure 6b exhibits the number of use reports per taxon that are further divided per use category. The 30 most significant taxa and their most popular uses are described below.

Remarkably, the most significant taxon identified is *Vitis vinifera*; it was found to be the most significant for all uses (Figure 6a), as well as for MED and CU uses, individually (Table S1). Despite the strong historical ties between Troodos inhabitants and winemaking [47,48], grapevine has been found to have a wide range of uses, including CU (96 use reports [URs]), MED (65 URs), and less for other uses (Figure 6b), indicating a particularly strong bond between local people and the taxon. Considering the MED uses, fruit products like the spirit ‘zivania’ has been used, e.g., against fevers/chills and as antiseptic, the molasse ‘epsima’ against constipation, and vinegar against insect bites, twig sap was used against eye discharge. Considering CU uses, its fruit products such as ‘epsima’ have been used in different sweets such as ‘retsellia’ and grape must (i.e., freshly pressed grape juice) has been used in sweets such as ‘soutzioukos’, ‘palouzes’, ‘kiofteri’, and ‘portos’ [49]. Grapevine leaves have been used in the cooked food ‘koupepia’ (i.e., vine leaves stuffed with ground meat, rice, and spices).

*Rosa damascena* (all uses second) has been utilized for several MED (40 URs), and CU uses (32 URs) and it is one of the few taxa considerably used in CO (16 URs) and religious traditions (RT; 6 URs). Its uses are mainly related to flower petals and their derived hydrolate ‘rodostema’ or ‘rodonero’ [49]. For example, ‘rodostema’ has been used against eyes discharge and for facial cleaning, and it is a main ingredient for several sweets such as ‘mahalepi’ and ‘palouzes’. ‘Rodostema’ has also been placed in ‘merreha’, a container with which the celebrants in RT were sprinkling the crowd. The petals have been used as a remedy for constipation, either consumed as spoon sweet or brewed into a tea.

The genus *Pistacia* (all uses third), represented in this study by *P. lentiscus* and *P. terebinthus*, has been mainly utilized for CU (33 URs) and MED purposes (14 URs). The fruits of *P. terebinthus* and, for some, those of *P. lentiscus* are used to prepare the pie ‘tremithopita’. Despite some common uses, the resin of *P. lentiscus* (mostly imported) mixed with olive oil has been used as liniment for treating chills/fever and general and abdominal pains. The fruits of *P. lentiscus* are used as an ingredient in smoked and salted cured pork meat sausages, and the fresh shoots of *P. terebinthus* are pickled for food.

*Rhus coriaria* (all uses fourth) was mainly utilized for tanning (17 URs) and traded primarily for that purpose (8 URs) [50]. However, it has been utilized also for MED (9 URs) and CU purposes (8 URs). The dried leaves were used to treat skin issues, such as skin pruritus or wounds. Its dried fruits have been used as a spice for cooked meat.

*Laurus nobilis* (all uses fifth) has been mainly utilized for CU (23 URs), MED (15 URs), and CO purposes (17 URs). Dried leaves have been used in cooked food (e.g., in ‘psito’ and ‘kleftiko’) as well as for food preservation (e.g., in ‘halloumi’, ‘trahanas’, and ‘elies tsakkistes’). *L. nobilis* herb-infused oil derived from fruits has been used against hair loss. In combination with other MAPs, such as *Myrtus communis* and *Rosmarinus officinalis*, the leaves of *L. nobilis* were used in a steam bath called ‘thermos’ to treat puerperium (i.e., the period of adjustment after childbirth) complications in women who have just given birth. Regarding CO uses, women used to use the fruits-derived oil for black hair dyeing and the leaves in boiled water for hair conditioning.

*Rosmarinus officinalis* (all uses sixth) has been mainly utilized for CU (24 URs), and MED purposes (18 URs). The aerial part of the plant was utilized mainly for food preservation in ‘zalatina’ (i.e., gelatin with meat pieces and spices [51]) and as ingredient in ‘savoro’ (i.e., marinated fish, sour sauce for preserving fish [51]). In addition to its use in ‘thermos’, the *R. officinalis* aerial part was used as tea; for example, against weakness and abdominal pain.

*Mentha spicata* (all uses seventh) has been mainly utilized for CU (45 URs), and MED purposes (27 URs). Concerning the CU uses, *M. spicata* leaves have been used as hot beverage (tea) and dried leaves as aromatic ingredient in ‘halloumi’ and ‘anari’ traditional cheeses, ‘flaounes’ (i.e., traditional eastern pies), ‘keftedes’ (i.e., meatballs), ‘koupepia’, ‘pastitsio’ (i.e., baked pasta with ground meat and béchamel sauce), and ‘apohtin’ (i.e., sun-dried salted goat meat). Regarding MED uses, *M. spicata* leaves have been used as tea especially against abdominal pain and other digestive complications such as dyspepsia.

The genus *Cistus* (all uses eighth), with main representative the *C. creticus*, has been mainly utilized for MED (27 URs), LC (14 URs), and CU purposes (10 URs). Regarding the MED uses, the leaves (as tea) have been used against several different issues, such as chills/fever, anxiety and weakness, elevated blood pressure, raised levels of cholesterol, and prostate symptoms, while its leaves’ resin has been used as liniment against general or abdominal pain or chills/fever in children. According to many respondents, *Cistus* resin was collected from the beards of the goats after foraging onto the oily foliage. Shepherds preferred *Cistus* plants for feeding their goats for high quality aromatic milk and, therefore, for ‘halloumi’ cheese production, and by beekeepers as a honey plant.

*Sambucus nigra* (all uses ninth) has been used mainly for MED (60 URs) and CU purposes (7 URs). Its flowers have been used as a hot beverage (tea). Concerning MED uses, *S. nigra* dried flowers have been mainly used against eyes discharge by decoction, while their tea against throat and other respiratory symptoms.

*Olea europaea* (all uses tenth) has been reported in this study mainly for MED purposes (40 URs). Olive oil has been used, among others, as liniment against infant colic and abdominal pain in children as well as herb-infused oil and in beeswax cream in combination with several of the other mentioned MAPs against different health issues, such as skin symptoms and abdominal pain.

*Origanum dubium* (all uses eleventh) has been mainly used for MED (36 URs) and CU purposes (18 URs). The dried leaves and flowers have been used as tea against various health issues such as chills/fever, respiratory and digestive issues and as herb-infused oil against, for example, skin issues. *O. dubium* has been used as a spice in several cooked foods (e.g., ‘souvla’) and as food preservative in ‘tsamarella’ (i.e., kind of goat boneless sun-dried meat).

The genus *Salvia* (all uses twelfth), with main representatives the *S. fruticosa* and *S. willeana* (Troodos endemic), has been mainly used for MED (24 URs) and CU purposes (18 URs). The most common use of *Salvia* or ‘spatzia’ leaves is tea. Troodos residents were used to drinking ‘spatzia’ tea daily, especially in the morning. The aerial part of the plant was used for smoking cured pork meat such as ‘loukanika’ (en. sausages) and ‘lountza’ (i.e., a kind of dried pork meat from skeletal muscles). Concerning the MED uses, *Salvia* leaves were consumed as tea against sweating problems, when feeling anxious or weak, for chills/fever, abdominal pain, and against memory disturbance.

*Myrtus communis* (all uses thirteenth) has been mostly utilized for MED (31 URs), and RT purposes (5 URs). Leaves were dried and powdered and used against diaper rush in infants. Except its use in ‘thermos’, *M. communis* leaves were used in foot bath or as powder against skin fungi infections. Regarding RT, people were welcoming important clerics, using *M. communis* aerial part.

*Lavandula angustifolia* (all uses fourteenth) has been mostly utilized for MED purposes (17 URs), and as animal repellent (9 URs). Flowers and leaves have been used as hydrolate against acne, headache, and sleep disturbance, as beeswax cream against insect bites, headache, and muscle pain, and as a tea against anxiety. Dried flowers have been used mainly as a clothes moth repellent.

*Allium cepa* (all uses fifteenth) has been mostly utilized for MED (21 URs), RT (10 URs), and CU purposes (5 URs). Its bulb was smashed and mainly applied as poultice on bruised parts of the body, such as knees. Its bulb outer scale leaves have been boiled for dyeing Easter eggs. Concerning CU uses, the onion bulb has been eaten raw or as an ingredient in cooked food (e.g., beans, ‘stifado’, i.e., rabbit stew with onions).

*Juglans regia* (all uses sixteenth) has been mainly utilized for MED (12 URs), MP (8 URs), CO (7 URs), and CU purposes (6 URs). Concerning CU uses, the fruit was used as a spoon sweet, the much served ‘karydaki’. The spoon-sweet-derived syrup was used mainly against mouth, tongue, or lip diseases. The leaves and/or fruit peels were boiled and used either for hair (black) dyeing or clothes dyeing (e.g., for ‘vraka’ the lower part of the traditional Cypriot male clothing).

*Prunus avium* (all uses seventeenth) has been mainly utilized for MED (30 URs) and CU purposes (11 URs). The dried fruit stalks (or pedicel) have been utilized as tea mainly against urological problems such as urinary retention or calculus, against prostate and kidney complaints. Concerning CU uses, the fruit has been used as a spoon sweet or marmalade.

The genus *Crataegus* (all uses eighteenth), with main representatives the species *C. azarolus* and *C. monogyna*, has been mainly utilized for MED (23 URs), and CU purposes (14 URs). MED uses have been mainly related to dried flowers used as tea against elevated blood pressure, heart issues, and increased cholesterol. The fruits have been mostly used to make a marmalade called ‘ladapi’, consumed also against digestive issues such as constipation.

The genus *Urtica* (all uses nineteenth), with several representatives in Cyprus [23], has been mainly exploited for MED (20 URs) and CU purposes (9 URs). Its leaves have been mainly an ingredient of salads, or eaten boiled, or ingredient of cooked foods (e.g., boiled with rice). Regarding the MED uses, fresh or dried leaves have been used to make tea against issues such as iron deficiency anaemia and urological issues, and the fresh leaves were used through rubbing against musculoskeletal issues such as joint issues and osteoarthrosis.

*Hypericum preforatum* (all uses twentieth), also called ‘valsamo’ (en. balsam) or ‘valsamochorto’ (en. balsam grass), has been mainly utilized for MED purposes (35 URs). The flowers and leaves were used to prepare an herb-infused oil or beeswax cream against many different medical issues such as skin (e.g., abrasions/cuts, burns) general pains, musculoskeletal (e.g., low back complaints, osteoarthrosis, muscle pain), psychological (e.g., feeling anxious, feeling depressed), and cardiovascular (e.g., varicose veins of leg).

*Prunus dulcis* (all uses twenty-first) has been mainly utilized for MED (15 URs) and CU purposes (7 URs). The fruit shell as tea has been used mainly against respiratory issues such as cough, sputum/phlegm, and throat complaint. The edible nut has been mainly used in sweets such as ‘soutzioukos’.

*Ocimum basilicum* (all uses twenty-second) has been mainly utilized for CU (8 URs), and MED purposes (6 URs). Regarding CU uses, its leaves have been used as an ingredient in spoon sweets and other traditional sweets such as ‘palouzes’. The leaves have also been used as tea against respiratory (e.g., pneumonia), psychological (e.g., feeling anxious) and neurological (e.g., headache) issues.

*Mentha longifolia* subsp. *cyprica* (all uses twenty-third) is an endemic plant and has been mainly utilized for CU (12 URs) and MED purposes (9 URs). Its fresh or dried leaves have been used as tea mainly against digestive issues, such as abdominal pain, and against chills/fever. Considering CU uses, the fresh leaves were eaten raw, in salads, or cooked with beans. Their foliage was also used to cover and preserve the apples.

*Matricaria chamomilla* (all uses twenty-fourth) has been mainly utilized for MED purposes (23 URs). The tea from its flowers has been mainly used against eye pain/eye discharge (in compresses) or digestive issues, such as abdominal pain in children or infant colic.

Two other species of the genus *Rosa* (all uses twenty-fifth), *R. canina* and the endemic *R. micrantha* subsp. *chionistrae*, showed different uses to *R. damascena*. They have been mainly utilized for MED purposes (16 URs). Their fruits have been mainly used to make tea against vitamin C deficiency or coughing.

*Pelargonium graveolens* (all uses twenty-sixth) has been mainly utilized for CU (18 URs), and MED purposes (5 URs). *Pelargonium* leaves have been mainly used as an important aromatic ingredient in sweets, such as ‘palouzes’ and several spoon sweets, and as tea against elevated blood pressure.

*Thymbra capitata* (all uses twenty-seventh) has been mainly utilized for CU (8 URs), and MED purposes (6 URs). Flowers and leaves have been used as a daily tea and against digestive issues, such as dyspepsia, against chills/fever and cough.

*Dittrichia viscosa* (all uses twenty-eighth) has been mainly utilized for MED purposes (12 URs). The leaves have been utilized against different skin (e.g., abrasions/cut, diaper rush in adults, localized swelling of legs), cardiovascular (e.g., haemorrhoids) and respiratory issues (e.g., cough, sputum/phlegm).

*Cydonia oblonga* (all uses twenty-ninth) has been mainly utilized for CU (17 URs) and MED purposes (12 URs). The fruit has been used as a spoon sweet, in a traditional sweet called ‘kydonopasto’, and as marmalade. Considering the MED purposes, the fruit peel as tea has been used against digestive issues such as diarrhoea.

*Pimpinella anisum* (all uses thirtieth) has been mainly utilized for MED (13 URs) and CU purposes (7 URs). Its seeds have been used as a daily tea and considering MED uses, mainly against abdominal pain, especially in children and infant colic.

### 2.6. Other Taxa, Exceptional and Fading Uses

In addition to the popular plants and uses previously mentioned, the study identified several other plants and plant uses that help to further elucidate the traditional relationships between people and plants in the mountainous area of Troodos.

Table S1 lists several additional taxa that were used to treat various MED issues. Some of them, according to the respondents, were used in mixtures, such as ‘thermos’, especially by women experienced in treating medicinal issues using traditional medicine and ‘iatrosophia’ (en. medical wisdom). Such occupations and treatments faded following the adoption of modern health care system in the island [42] as well as in the study area according to the respondents. Other taxa have been used mainly for specific issues and were, therefore, not included in the most significant taxa. For example, the leaves of ‘lithospastos’ (en. breaking stones), the species *Plantago coronopus*, have been used mainly as tea against urinary calculus. The leaves of the genus *Eucalyptus*, with several representatives in Cyprus [19] and especially the *E. citriodora*, have been used mainly in steam bath against respiratory issues. The tubers of *Asphodelus ramosus* and the bulbs of *Drimia aphylla* have been each smashed and applied as poultices on skin abrasions/cuts, blisters, and bruises. The tubers of *Solanum tuberosum* have also been applied in the same way against burns. The seeds of *Papaver somniferum* were used in the past, but not anymore, as tea for children feeling anxious.

A large proportion of the CU uses mentioned were attributed to sweets and desserts as well as food preservation. Making spoon sweets or marmalades was a way to preserve different fruits (e.g., *Citrus × aurantium*, *Prunus avium*, *Malus domestica*, *Prunus persica*, *Cydonia oblonga*, *Ficus carica*, *Citrullus lanatus*, *Citrus x sinensis*, *Pyrus communis*, *Juglans regia*, *Crataegus* spp.) using syrup and ingredients from other plants (e.g., *Pelargonium graveolens*, *Rosa damascena*, *Syzygium aromaticum*, *Cinnamomum verum*, *Pinus* spp.) for seasons in which fresh fruits were not available. Each fruit provides a distinct aroma and taste to the spoon sweet. The same principle has been applied for pickled food using vinegar to preserve young shoots, fruits, leaves, or flower buds of other taxa (e.g., *Pistacia terebinthus*, *Eryngium creticum*, *Capparis spinosa*). Several taxa, such as *Foeniculum vulgare*, *Origanum dubium*, *Rosmarinus officinalis*, *Origanum majorana*, *Cistus spp.*, *Coriandrum sativum*, *Allium sativum.*, *Piper nigrum*, *Allium cepa*, *Apium graveolens*, *Cuminum cyminum*, *Citrus x aurantium*, and *Laurus nobilis*, have been used as ingredients in preserved food. Other taxa have been used as raw food for their fruits (e.g., *Myrtus communis*, *Rubus sanctus*, *Berberis cretica*, *Arbutus andrachne*, *Rosa* spp., *Castanea sativa*, *Corylus* spp., *Elaeagnus angustifolia*, *Mespilus germanica*), leaves and shoots in salads or by themselves (e.g., *Mentha longifolia*, *Mentha spicata*, *Silene vulgaris*, *Nasturtium officinale*, *Plantago major*, *Taraxacum* spp., *Helosciadium nodiflorum*, *Ocimum basilicum*, *Cynara* spp.) or stems (e.g., *Echinops spinosissimus*). Even though the respondents did not refer to a specific medicinal or aromatic use for several of the aforementioned taxa, they believed that their consumption had health benefits. While the traditional MED uses of many taxa mentioned above (*Allium cepa*, *Taraxacum* spp., etc.) are well recognized worldwide [52,53], the lack of specified use inevitably indicates that their traditional uses are clearly fading.

Despite the MED and CU uses, taxa have had or continue to have some more uses in Troodos. For example, the dried aerial part of *Thymbra capitata* and *Origanum majorana* were used as brooms. *Rhus coriaria*, *Juglans regia*, *Rhamnus alaternus*, *Punica granatum*, *Glebionis coronaria* and *Papaver rhoeas* have been used in dyeing threads or clothes. The recollection by elders of the use of the plant (*R. coriaria*) in the past sold as a dye was also recorded in a study in Sicily [11]. *Euphorbia veneris*, *Verbascum sinuatum*, *and Styrax officinalis* were used for eels fishing. The resin of *Pinus spp*. was used for wineskin waterproofing. The cladode sap of *Opuntia ficus—indica* was added to asbestos paint for stabilization. *Vicia dalmatica*, *Rosmarinus officinalis*, *Thymbra capitata*, *Cistus* spp., *Melissa officinalis* and others have been considered honey plants of high importance. *Vicia dalmatica* has also been considered as a health and yield promoter when present in vineyards and nutritious forage for goats and donkeys. *Erophaca baetica* have been considered poisonous for goats and *Nerium oleander* poisonous for bees and other animals. *Dittrichia viscosa* has been considered as a repellent for bee and plant parasites. *Ocimum basilicum* and *Pelargonium graveolens* were used for religious water blessing and *Olea euroaea* leaves as incense. Finally, branches and trunks of different species, such as *Vitis vinifera*, *Pistacia* spp., *and Laurus nobilis* have been burned and the ash was mixed with water to prepare their laundry detergent called ‘alousiva’. ‘Alousiva’ was used as a laundry detergent but also for bathing.

Even though some uses are fading in time because of several reasons, it seems that the traditional knowledge on MAPs uses in Troodos, based on the number of the use reports (Figure S2a) and the number of taxa mentioned (Figure S2b), is not declining as the respondents’ age decreases, at least within the age range of the respondents in this study. This is an indication of an active knowledge transmission between generations in the mountainous area of Troodos. However, the factors previously mentioned do not necessarily ensure the preservation and transfer of the current knowledge and understanding of the traditional MAPs uses in the Troodos region.

## 3. Materials and Methods

### 3.1. Study Area

The study was conducted in the Troodos Mountain Range (Figure 1b), within the second phytogeographical division of Cyprus [21,22]. The study area encompasses mainly mountainous communities within or adjacent to the second phytogeographical division (Figure 1c). The 31 communities are well dispersed within the region covering a substantial area (~640 km^2^) of the second phytogeographical division (~1794 km^2^).

### 3.2. Sample Survey & Data Collection

To collect the primary information, semi-structured face-to-face interviews were conducted in 2019 and 2020 with 57 respondents in 40 groups. Twenty-nine of these were individual interviews, and eleven were group interviews with twenty-eight respondents in total. The respondents were local people from the 31 Troodos communities (Figure 1c) who possessed extensive knowledge of traditional MAPs uses. Some of the informants can be characterized as parataxonomists, i.e., “people who identify biological samples without having had formal training in taxonomy and systematics” [54]. Regarding the sampling method, exponential discriminative snowball sampling was used to identify and select the participants [55]. This is a useful approach when there is difficulty in identifying members of interest within a population and/or the time and financial resources required are limited [56], as was the case with this study. Using this method, the research team initially identified some suitable key informants. After the completion of interviews, they were asked to suggest other potential participants relevant to the respective research, who in turn suggested others, and so on [56]. This sequential sampling method resulted in an adequate number of respondents (sample size) to gather the required information. The data saturation stopping rule was applied to stop sampling [57]. It is important to note that the final decision on the selection (or non-selection) of the participants was made by the research team, considering the research objectives and limitations. The information collected during the interviews was primarily related to the traditional uses of MAPs in the region and the socio-demographic characteristics of the participants. In each interview, the first two authors served as moderators, whilst an interview guide was prepared and used to assist the interview process. In addition to the notes taken by the researchers, all interviews were audio taped and transcribed verbatim to ensure data accuracy; a permission was granted by the interviewees in advance. Each interview lasted ca. two hours.

The collected information was tabulated into Microsoft Excel (Office 365) to establish a database. Each row in the data sheet was considered as one use report, and the information for each use report was divided in columns, as follows: respondent’s group number, respondent’s number, plant family, taxon, scientific name, vernacular name(s), use category (see below in Section 3.3.1, e.g., MED for medicinal), use sub-category (e.g., digestive), specific use (e.g., abdominal pain), plant part used, form of preparation, form of application, and plant status (i.e., collected or cultivated).

Most of the plant taxa were identified in the field using, when necessary, the online “Flora of Cyprus—a dynamic checklist” [23] and the “Flora of Cyprus” Volume 1 and 2 [21,22]. A few taxa were identified by cross-checking the respondents’ references with botanical experts in the field. The taxa scientific names that are listed in this research follow the nomenclature of the Flora of Cyprus checklist [23]. This database is frequently updated (latest update 15 February 2023) and follows the latest internationally accepted nomenclature of the Kew Royal Botanic Gardens [58] and Euro+Med Plant Base [59]. For a few cultivated taxa that are not included in the Flora of Cyprus, the nomenclature follows the International Plant Names Index [60] and Euro+Med Plant Base [59]. Vernacular names in Greek were converted to Latin characters following the GrElot 743 standards [61].

### 3.3. Quantitative Ethnobotany

Quantitative ethnobotany is a research approach that employs quantitative methods to study the utilization of plants by different cultures or communities [62]. This approach, employed in this study, involves direct analysis of data on plant uses to better understand the ways in which people interact with their surrounding plant life.

#### 3.3.1. Use Categories

Uses’ classification and categories’ description is essential for calculating certain ethnobotanical indices (see below in Section 3.3.2) and studying the diversity of the MAPs traditional uses. The general uses of MAPs were classified into 12 categories, based on the outcomes of the interviews and the relevant literature [12,13]: medicinal (MED), culinary (CU), cosmetics (CO), livestock care (LC), crop care (CC), religious traditions (RT), manufacturing—processing (MP), household uses (HU), trade (TR), animal repellent (AR), fishing–hunting (FH), and poisonous plants (PP).

Medicinal (MED) is defined as the use of plants for their therapeutic properties and pharmacological effects on the human body [63]. For the purposes of the current study, medicinal uses were further classified into 16 sub-categories according to the International Classification of Primary Care (ICPC-2) [64], a classification system acknowledged by the World Health Organization. The ICPC-2 classification was adopted since it is more consistent with ethnomedical reality than other classification systems, e.g., the International Classification of Diseases or the Economic Botany Data Collection Standard, as the categories are designed according to patient’s perceptions and are less influenced by clinical medicine [65]. The MED sub-categories formed are: general and unspecified (A), blood, blood forming organs and immune mechanism (B), digestive (D), eye (F), ear (H), cardiovascular (K), musculoskeletal (L), neurological (N), psychological (P), respiratory (R), skin (S), endocrine/metabolic and nutritional (T), urological (U), pregnancy, childbearing (W), female genital (X), and male genital (Y).

Culinary (CU) is defined as the use of plants in foods and beverages for human consumption. CU uses were further classified based on the participants’ responses to the following 13 sub-categories: hot beverages (e.g., tea), cold beverages, alcoholic beverages (or ingredients for alcoholic beverages and/or plants used in the preparation process of alcoholic beverages), sweets and desserts, food preservation (or ingredients of preserved food), raw food (i.e., eaten raw), dried fruits, herbs and spices (i.e., aromatic plant material in small quantities to flavor food), pickled food, cooked food ingredient (large portions), non-cooked food ingredient (large portions, e.g., salads), condiment–other (e.g., vinegar, juice, oil), and smoking flavoring (i.e., plants used in the process of flavoring and preserving food).

Cosmetic use (CO) is defined as the application of plants’ parts or preparations on external parts of the human body, such as the skin, hair, nails, lips, and external genitalia, or on the teeth and the mucous membranes of the oral cavity, with the main purpose of cleaning, perfuming, altering appearance, eliminate body odor, and/or protecting and maintaining a good condition [66].

Livestock care (LC) is defined as the use of plants in livestock diet (i.e., forage), and for specific dietary needs, as well as for veterinary practices. This category also includes honey (beekeeping) plants.

Crop care (CC) is defined as the use of plants to protect crops from biotic agents (e.g., parasitic insects), enhance crop yield, address soil erosion, protect cultivated plants and their products from abiotic factors, and promote the overall health of the crops and their fruits.

Religious traditions (RT) is defined as the use of plants in religious traditions. Religious traditions are constellations of beliefs, practices, and institutions used to describe a common type of religiosity [67].

Manufacturing—processing (MP) is defined as the use of plants in manufacturing or processing of materials. Manufacturing is defined as the development of new products from raw materials (e.g., baskets, brooms, earthenware jars, toys), while material processing is defined as the operations that transform a raw material into an end-product (e.g., dyeing, painting, tanning).

Household uses (HU) is defined as the use of plants for household needs (e.g., in laundry, as kindling/fuel wood, aromatics for closets).

Trade (TR) is defined as the use of plants for trading purposes, either domestically or for exports. Respondents indicated that some plants in this category were not used locally but exclusively traded, while other plants (or plants’ material) were used locally but also exported.

Animal repellent (AR) is defined as the use of plants or plant material as animal repellents (e.g., human pests, snakes, rodents).

Fishing–hunting (FH) is defined as the use of plants or plant material for fishing or hunting purposes.

Poisonous plant (PP) is defined as a plant that when touched or ingested in sufficient quantity can be harmful or fatal to an organism, or any plant capable of evoking a toxic and/or fatal reaction [68].

#### 3.3.2. Ethnobotanical Indices

The six ethnobotanical indices used are described below, along with the respective equations applied. For the selection of the indices, a comprehensive list of cultural importance indices was reviewed [43,44,45]. The selection was based on their widespread use in previous studies and their relevance to the specific objectives of this research. The indices were initially calculated for all use categories. Subsequently, for MED and CU uses, the indices were also computed separately, and their sub-categories were treated as categories when computing indices that require the number of categories.

1. Use Reports (*UR_S_*): A widely applied metric [17,42,45] used to study the cultural importance of plants is the total number of use reports (*UR*) for each taxon (*UR_S_*). Every use of a taxon reported in each one of the interviews is counted as one *UR*. Reports referring to identical uses of a taxa from the same informant or group of informants (when applicable) were removed from the database to avoid duplicates. Following Kufer et al. [69], *UR* is defined as:
  (1) $$UR_{s}=\sum_{u=u_{1}}^{u_{nc}}\sum_{i=i_{1}}^{i_{n}}UR_{ui}\;$$
  where:
  *nc*: is the total number of use categories (*u*), and
  *n*: the total number of informants
  First, the *UR* of all the informants (from *i*_1_ to *i_n_*) within each use category for that taxon (*s*) were summed, i.e., the number of informants who mention each use category for the taxon. Second, all the *UR* of each use category (from *u*_1_ to *u_nc_*) were summed.
2. Use Value (UV_S_): Indicates the taxa which are considered most important by a given population. High UVs values imply that a plant taxon is important. However, UVs does not give information on the versatility of taxa uses. UV_S_ was proposed by Phillips and Gentry [70], simplified by Rossato et al. [71] and Albuquerque et al. [72], thoroughly explained by Tardio and Pardo-De-Stanayana [45], and is calculated as:
  (2) $${\mathrm{UV}}_{s}=\frac{UR_{s}}{n}$$
  where:
  *UR_s_*: the total use reports for a taxon, and
  *n*: the total number of informants’ groups considering that all informants participated in the survey were interviewed for all the taxa.
3. Relative Frequency of Citation (*RFC_S_*): Measures the plants that were the most frequently mentioned as useful. The index does not account for the variable use category [45]. This index scores range from 0, when nobody refers to the plant as useful, to 1 in the improbable case that all the informants would mention the use of the taxon. It is computed as [45]:
  (3) $$RFC_{s}=\frac{FC_{s}}{n}$$
  where:
  *FC_s_*: the number of informants’ groups who mention the use of the taxon, also known as frequency of citation, and
  *n*: the total number of informants’ groups.
4. Relative Importance (*RI_S_*): *RI_S_* is a measure of the versatility of the uses. Taxa with high *RI* values (near 2) are the most diversified in terms of uses [17]. It was proposed by Bennet and Prance [73] and adapted by Albuquerque et al. [72], as follows:
  (4) $$RI_{S}=UC_{S/VS}+USC_{S/VS}$$
  (5) $$UC_{S/VS}=\frac{UC_{S}}{UC_{VS}}\;$$
  (6) $$USC_{S/VS}=\frac{USC_{S}}{USC_{VS}}$$
  where:
  *UC_s_*_/*vs*_: the number of use categories of a given taxon (*UC_S_*) divided by the total number of use categories of the most versatile taxon (*UC_VS_*) (Equation (5)), and
  *USC_s_*_/*vs*_: the total number of types of uses or groups attributed to a given taxon (*USC_s_*), irrespective of the use category, divided by the total number of types of uses or groups attributed to the most important taxon, (*USC_vs_*; Equation (6)), independent of the number of informants that cite the taxon.
5. Relative Importance Index (*RII_S_*): This index theoretically varies from 0, when nobody mentions any use of the plant, to 1 in the improbable case that all the informants mention the use of the taxon in all the use categories. It was proposed by Pardo de Santayana [74] and thoroughly explained by Tardío and Pardo-De-Santayana [45], as follows:
  (7) $$RII_{s}=\frac{RFC_{s\left(\max\right)}+RNU_{s\left(max\right)}}{2}$$
  (8) $$RFC_{s\left(\max\right)}=\frac{FC_{s}}{\max\left(FC\right)}$$
  (9) $$RNU_{s\left(\max\right)}=\frac{NU_{s}}{\max\left(NU\right)}$$
  where:
  *RFCs*_(max)_: the FC_S_ over the maximum *FC*, obtained by dividing *FC_S_* by the maximum *FC* value in all surveyed taxa (Equation (8)), and
  *RNU_s_*_(max)_: the relative number of use categories (*NU_S_*) over the maximum *NU*, obtained by dividing the number of uses of the taxon by the maximum value in all the taxa of the survey (Equation (9)).
6. Cultural Value Index (*CVs*): Is a function of both the number of the uses and the number of respondents’ groups reporting the plant. It was proposed by Reyes-Garcia et al. [75] and explained by Tardio and Pardo-De-Santayana [45], as follows:
  (10) $$CV_{s}=\left[\frac{UC_{S}}{UC_{total}}\right]\times \left[\frac{FC_{s}}{n}\right]\times \left[\frac{UR_{s}}{n}\right]$$
  where:
  the first factor is the relationship between the number of different uses reported for the species (*UC_s_*) and the total number of use categories considered in the study (*UC_total_*); the second factor is the relative frequency of citation of the species (as previously defined); the third factor is the sum of all the use reports for the taxon divided by *n* (as previously defined).

The consensus in information related to the use categories as well as the MED and CU sub-categories was evaluated by the Informant Consensus Factor (*ICF*) [76]:

(11) $$ICF=\frac{N_{ur}-N_{t}}{N_{ur}-1}$$

where:

*N_ur_* is the number of use reports for a use category (or sub-category), and
*N_t_* is the number of taxa used for the particular use category (or sub-category).

The value of this factor varies from 0 to 1. A high value indicates that relatively few taxa are reported by a high number of respondents, while a low value indicates that the informants disagree on the taxa to be used, for instance, within a use category or a MED issue or a CU use.

### 3.4. Statistics

Spearman’s correlation coefficient was employed to evaluate the monotonic relationship between the six selected indices for different plant taxa. The ethnobotanical indices were calculated based on general uses reported, and solely for MED and CU uses. Correlation matrices were constructed to summarize and visualize associations between the selected indices.

## 4. Conclusions

The present research is the first ethnobotanical assessment for the Troodos Mountains in Cyprus, focusing not just on medicinal and aromatic plant uses, but also identifying a wide range of other uses. The comparison between six ethnobotanical indices indicated that the cultural value index [75] was the most suitable index for evaluating cultural significance. The study has added to the understanding of the diverse uses of plants among the local people of Troodos and revealed a deep connection (e.g., cultural, economic, and spiritual) between these people and the plants of the area. Many of the plants identified in the study have been mentioned in previous studies at both the national level [17,37], the wider Mediterranean region [9], in Mediterranean [77] and Balkan mountainous areas [78], as well as in historical studies [42,79,80]. Most of the uses identified have been maintained due to the isolation of the population and their desire to preserve their traditions, while some have faded away due to changing lifestyles. As a result of this study, in 2022 the *Traditional Uses of MAPs in the Mountainous Area of Troodos* have been inscribed as an element of the Intangible Cultural Heritage of Cyprus by the Cyprus National Commission for UNESCO. The respondents of the study expressed a strong preference for sustainable living and the importance of conservation of culture and nature.

## Figures and Tables

**Table 1:** Demographic characteristics (respondents = 57).

Parameter		Value
Gender	Female	54.4%
	Male	45.6%
Age	30–49	7.0%
	50–69	28.1%
	70–89	54.4%
	≥90	10.5%
	Average	73 years
Education	Primary	33.3%
	Secondary	40.4%
	Tertiary	26.3%

## Data Availability

Data sharing is not applicable to this article.

## Supplementary Materials

The following supporting information can be downloaded at: https://www.mdpi.com/article/10.3390/plants12051119/s1, Figure S1: The Informant Consensus Factor (ICF) for the use categories, the medicinal, and culinary sub-categories; Table S1: The plant taxa, recorded in the present ethnobotanical study; Figure S2: The relationships of the use reports and the number of taxa mentioned with the average respondents’ age.
