# Supplementary material for: An Ethnobotanical Investigation into the Traditional Uses of Mediterranean Medicinal and Aromatic Plants: The Case of Troodos Mountains in Cyprus
Source: Plants (Basel). 2023 Mar 2;12(5):1119. doi: 10.3390/plants12051119 (PMC10005048; doi:10.3390/plants12051119)
Supplement: Supplementary file 1 [file plants-12-01119-s001.zip › plants-2209146-supplementary.pdf]

Supplementary Material

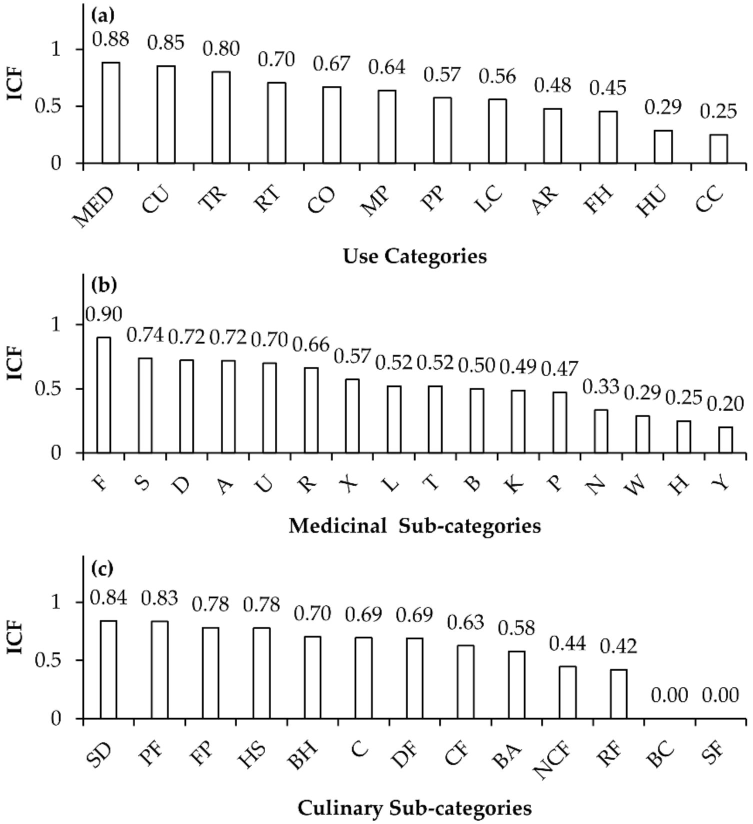

Figure S1. The Informant Consensus Factor (ICF) for the use categories* (a), the medicinal** (b), and culinary sub-categories*** (c). *Use categories: Medicinal (MED), Culinary (CU), Livestock care (LC), cosmetics (CO), manufacture-processing (MP), religious traditions (RT), animal repellent (AR), crop care (CC), household use (HU), fishing-hunting (FH), trade (TR), poisonous plants (PP). **Medicinal sub-categories (according to ICPC-2): Skin (S), digestive (D), general & unspecified (A), respiratory (R), eye (F), cardiovascular (K), urological (U), psychological (P), musculoskeletal (L), endocrine/metabolic & nutritional (T), pregnancy, childbearing (W), neurological (N), blood, blood forming organs and immune mechanism (B), female genital (X), male genital (Y), ear (H). *** Culinary sub-categories: Sweets & desserts (SD), food preservation (FP), herbs & spices, (HS), hot beverage (tea; BH), cooked food ingredient (CF), raw food (RF), alcoholic beverage (BA), non-cooked food ingredient (NCF), dried fruits (DF), condiment (other; C), pickled food (PF), cold beverage (BC), smoke flavoring (SF).

Table S1. The plant taxa, recorded in the present ethnobotanical study, are organized alphabetically by plant division, family, and genus. Information is provided on whether the taxa are indigenous or endemic [23] and whether are collected, cultivated, or imported according to the respondents, the vernacular names recorded, the use categories, the medicinal and culinary sub-categories in which their uses were classified, and their ranking according to the Cultural Value Index when calculated considering all, medicinal and culinary uses respectively. (--: no information, the abbreviations are clearly defined below the table* and thoroughly explained in section 2.3.1).

Taxon, Family, Indigenous (IN)/Endemic (EN), Collected (co)/cultivated (cu)/imported (im)	Vernacular name(s)	Use Categories	CV Rank (All)	Med Sub-categories	CV Rank (Med)	Cu Sub-categories	CV Rank (Cu)
**Pteridophyta**							
Pteridium aquilinum (L.) Kuhn, Dennstaedtiaceae, IN, co	Fteritzin	MED	122	S	78		
Equisetum spp., Equisetaceae, IN, co	Polykompi	MED, CC	90	A, L, S, T, U	47		
Adiantum capillus-veneris L., Pteridaceae, IN, co	Polytrichi, skorpidi	MED	108	S	67		
**Spermatophyta**							
Sambucus nigra L., Adoxaxeae, --, co/cu	Zampoukos, koufoxylia	MED, CU, CC	9	A, D, F, K, P, R, S	2	BC, BH	29
Amaranthus spp., Amaranthaceae, --, co	Glintos	CU	122			CF	71
Allium ampeloprasum L., Amaryllidaceae, IN, co	Skouraththa, agrio prasso	CU, LC	69			CF	51
Allium cepa L., Amaryllidaceae, --, cu	Kremmydi	MED, CU, RT, MP	15	L, R, S	19	CF, FP, RF	34
Allium sativum L., Amaryllidaceae, --, cu	Skordos, skortos, skordo	MED, CU, AR, CC	38	A, D, H, K, N	29	FP	64
Pistacia spp., Anacardiaceae, IN, co/im	Mastichodentro, ssinia, ssinos (*P. lentiscus*) ; tremithkia, trimithkia, tremithia (*P. terebinthus*)	MED, CU, LC, Co, MP, HU, CC, TR	3	A, D, S	28	C, DF, CF, FP, PF, RF, HS	2
Rhus coriaria L., Anacardiaceae, IN, co	Roudin, routhkia, soumatzin, soumaki	MED, CU, LC, Co, MP, CC, FH, TR	4	A, D, S	31	HS	33
Anethum graveolens L., Apiaceae, --, cu	Anithos, anitho	CU	122			BA	71
Apium graveolens L., Apiaceae, IN, cu	Sellino, selleno	MED, CU	81	B	78	CF, FP	60
Coriandrum sativum L., Apiaceae, --, cu	Koliantros	CU	48			FP	23
Cuminum cyminum L., Apiaceae, --, cu/im	Artisia	CU	92			FP, HS	44
Eryngium creticum Lam., Apiaceae, IN, co	Pagkallos, magkallos	CU	89			CF, PF	42
Foeniculum vulgare Mill., Apiaceae, IN, co	Maratho, marathos	MED, CU	31	A, D, P, U	37	BH, CF, FP, HS, SD	6
Helosciadium nodiflorum (L.) W. D. J. Koch, Apiaceae, IN, co	Sellinoui tou potamou, arkoselleno	CU	122			NCF	71
Petroselinum crispum (Mill.) A. W. Hill, Apiaceae, --, cu	Maitanos	CU	108			HS	64
Pimpinella anisum L., Apiaceae, --, cu/im	Glykaniso, glykanisos	MED, CU	30	A, D, R	25	BA, BH	30

Smyrnium olusatrum L., Apiaceae, IN, co	Arkoselleno	CU	122			CF	71
Nerium oleander L., Apocynaceae, IN, co	Arodafni, pikrodafni	MED, PP	85	S	67		
Hedera pastuchovii subsp. *cypria* (McAllister) Hand, Araliaceae, EN, co	Kissos	MED	108	S	67		
Asparagus acutifolius L., Asparagaceae, IN, co	Agrellin, agrellia	CU	95			CF	55
Drimia aphylla (Forssk.) J. C. Manning & Goldblatt, Asparagaceae, IN, co	Avrossillos, avrossilla, skylla, skyllokremmyda, vavitsa	MED, LC, MP, AR, PP	41	K, S	41		
Ruscus aculeatus L., Asparagaceae, IN, co	--	MED	122	L	78		
Achillea spp., Asteraceae, --, co	Achilleia	MED	90	A, K, X	42		
Artemisia arborescens L., Asteraceae, --, co	Genia tou gerou	MED	108	A, D	59		
Calendula officinalis L., Asteraceae, --, cu	Katifes, kalentoula	MED, AR	81	K, S	59		
Carthamus tinctorius L., Asteraceae, --, cu	Zacharas, zafaras	CU	78			HS	47
Cichorium intybus L., Asteraceae, IN, co	Kichorio, agrio radiki	MED	122	T	78		
Cynara spp., Asteraceae, IN, co	Agrioagkinara (*C. cornigera*), chosti (*C. cardunculus*), aplotarka	MED, CU	50	D, K	51	CF, RF	36
Dittrichia viscosa subsp. *angustifolia* (Bég.) Greuter, Asteraceae, IN, co	Konyzos	MED, LC, AR, CC	28	A, K, P, R, S	21		
Echinops spinosissimus Turra, Asteraceae, IN, co	Kefalagkathos, kamilagkathos	MED, CU, LC	80		105	RF	71
Glebionis coronaria (L.) Spach, Asteraceae, IN, co	Similoudin, kitrini margarita, papouna, lazaros, agrio sellino	MED, CU, RT, MP	33	R	76	CF, RF	44
Helichrysum italicum (Roth) Don, Asteraceae, IN, co	Psyllina, psyllida	MED, CU, HU, CC	47	S	78	BA, FP	48
Matricaria chamomilla L., Asteraceae, IN, co	Chamomili, mougiochorto, papouna	MED, CU, Co, AR	24	A, D, F, K, P, R, S, X	12	BH	55
Onopordum cyprium Eig, Asteraceae, EN, co	Gaidouragkatho	MED	76	D	50		
Sonchus oleraceus L., Asteraceae, IN, co	Galatouna	MED, CU	99	D	78	CF	71
Tanacetum balsamita L., Asteraceae, --, co	Valsamo	MED	105	A, N, S	53		
Taraxacum spp., Asteraceae, --, co	Agrioradiko	MED, CU	64	D, U	54	NCF	70
Berberis cretica L., Berberidaceae, IN, co	Ververissia, agkathovatos	CU, MP	99			RF	71
Corylus spp., Betulaceae, --, cu	Fountoukia	CU	122			RF	71
Anchusa azurea Mill., Boraginaceae, IN, co	Vouglosson, mouglosson	MED, CU	99	D	78	CF	71
Brassica oleracea var. *botrytis*, Brassicaceae, --, cu	Kounouppidi	CU	122			CF	71
Nasturtium officinale W. T. Aiton, Brassicaceae, IN, co	Kardamilla, kartamilla, kardamo, nerokardamo	CU	71			CF, NCF, RF	28
Sinapis alba L., Brassicaceae, IN, co	Lapsana	CU, LC	81			CF, NCF	60
Opuntia ficus-indica (L.) Mill., Cactaceae, --, co	Papoutsosytzia	MED, MP	81	S	78		
Cannabis sativa L., Cannabaceae, --, cu	Kannaourin	CU	108			RF, HS	60
Capparis spinosa L., Capparaceae, IN, co	Kapparka, kappari	CU, LC	52			PF, RF	27
Silene vulgaris (Moench) Garcke, Caryophyllaceae, IN, co	Strouthkia, stroufouthkia, tsakrithkia	CU	57			CF, NCF	22

Cistus spp., Cistaceae, --, co	Xistarka, ladania, katsarka	MED, CU, LC, Co, HU, TR	8	A, D, K, N, P, R, S, T, Y	7	BH, FP, SF	15
Citrullus lanatus (Thunb.) Mansf., Cucurbitaceae, --, cu	Karpouzia	CU	122			SD	71
Cucurbita moschata Duch., Cucurbitaceae, --, cu	Kotsinokoloko	CU	122			SD	71
Cucurbita pepo L., Cucurbitaceae, --, cu	Kolokythia	MED, CU	77	A, N,	55	SD	71
Ecballium elaterium (L.) A. Rich, Cucurbitaceae, IN, co	Pikrangouria	MED	122	A	78		
Cupressus sempervirens L., Cupressaceae, IN, co	Kyparissi	MED	122	S	78		
Juniperus spp., Cupressaceae, IN, co	Aoratos	LC, AR	99				
Cytinus hypocistis (L.) L., Cytinaceae, IN, co	Mitzilia, vizilia, vizinia	CU	95			RF	55
Elaeagnus angustifolia L., Elaeagnaceae, --, co	Zizifia	MED, CU	58	R	63	RF	51
Arbutus andrachne L., Ericaceae, IN, co	Antrouklia, koumaria	MED, CU, LC, FH	36	A, P	59	BA, RF, SD	31
Euphorbia veneris M. S. Khan, Euphorbiaceae, EN, co	Tsounna	FH	122				
Acacia saligna (Labill.) H. Wendl., Fabaceae, --, co	Akakia	LC	122				
Ceratonia siliqua L., Fabaceae, IN, cu	Teratsia, charoupia	MED, CU, HU	60	R,	67	BA, SF	64
Cicer arietinum L., Fabaceae, --, cu	Revithi	CU	122			CF	71
Erophaca baetica subsp. *orientalis* (Chater & Meikle) Podlech, Fabaceae, IN, co	Pifanis, pellopifanos, arkokoutzia, agriokoukia	LC, PP	66				
Glycyrrhiza glabra L., Fabaceae, IN, co	Glykoriza	MED	122	D	78		
Lens culinaris Medik., Fabaceae, --, cu	Fatzii	CU	122			CF	71
Pisum sativum L., Fabaceae, IN, cu	Pizeli	LC	122				
Vicia dalmatica A. Kern., Fabaceae, IN, cu	Mavrassieron	MED, CU, LC, CC	39	D	78	BH	71
Vicia ervilia (L.) Willd., Fabaceae, IN, cu	Rovi, rovassiero	LC	108				
Vicia faba L., Fabaceae, --, cu	Koutzia	LC	122				
Castanea sativa Mill., Fagaceae, --, cu	Kastania	CU	122			RF	71
Quercus alnifolia Poech, Fagaceae, EN, co	Latzia	MED, CU, LC, MP, FH	40	W	78	CF	71
Quercus coccifera subsp. *calliprinos* (Webb) Holmboe, Fagaceae, IN, co	Pernia	MED, CU, LC, MP	67	S	78	RF	71
Quercus infectoria subsp. *veneris* (A. Kern.) Meikle, Fagaceae, IN, co	Valanidia, drys	CU, LC, HU	49			RF	71
Geranium tuberosum L., Geraniaceae, IN, co	Pirpillina	CU	122			RF	71
Pelargonium graveolens (Thunb.) L'Hér., Geraniaceae, --, cu	Kiouli, armparoriza	MED, CU, RT	26	K, P, S, W	40	BH, FP, HS, SD	7
Hypericum perforatum L., Hypericaceae, IN, co/cu	Yperiko, spathochorto, valsamochorto, valsamo	MED, CU, Co, CC	20	A, D, K, L, N, P, S, Y	9	BH	71

Juglans regia L., Juglandaceae, --, cu	Karydia, karythkia	MED, CU, LC, Co, MP	16	D, K, R, S, W	24	RF, SD	34
Calamintha incana (Sm.) Benth., Lamiaceae, IN, co	Glifoni	MED	122	S	78		
Lavandula angustifolia Mill., Lamiaceae, --, cu	Levanta	MED, CU, Co, RT, AR, HU, CC	14	A, D, L, N, P, S	20	BH	55
Lavandula stoechas L., Lamiaceae, IN, co	Myrofora, agria levanta	LC, RT	108				
Melissa officinalis L., Lamiaceae, IN, co	Melissochorto	MED, CU, LC	55	B, K, P, S	36	BH	71
Mentha aquatica L., Lamiaceae, IN, co	Vasilikodyosmos, fliskouni	MED	122		105		
Mentha longifolia subsp. *cypricа* (Heinr. Braun) Harley, Lamiaceae, EN, co	Potamoeitanos, potamogeitonas, agria menta	MED, CU, Co, AR	23	A, D, N, P	27	BH, NCF, FP, RF, HS	12
Mentha spicata L., Lamiaceae, IN, cu	Dkyosmis, dyosmos	MED, CU, HU	7	A, D, N, P, S	8	BH, NCF, FP, HS	3
Mentha x *Piperita* L. , Lamiaceae, --, cu	Menta	MED	122	D	78		
Micromeria chionistrae Meikle, Lamiaceae, EN, co	--	Co	122				
Ocimum basilicum L., Lamiaceae, --, cu	Vasilikos, vasilitzia	MED, CU, RT, AR, HU, CC	22	D, N, P, R, S	33	BH, NCF, HS, SD	25
Origanum dubium Boiss., Lamiaceae, nEN, co/cu	Rigani, rigani tou Kampou	MED, CU, LC, Co, CC	11	A, D, L, R, S, U	6	BH, FP, HS	9
Origanum majorana var. *tenuifolium* Weston, Lamiaceae, EN, co	Sapsisia, matzourana	MED, CU, Co, MP	34	A, D, L, R, S, U, W	32	FP, HS	51
Rosmarinus officinalis L., Lamiaceae, IN, co,cu	Lasmaris, lasmari, dentrolivano	MED, CU, LC, Co, RT, AR	6	A, D, K, P, S, T, W	15	BH, FP, HS	5
Salvia spp., Lamiaceae, IN, co	Aspatzia, spatzia, chachomilia, faskomilo	MED, CU, Co, RT, AR	12	A, D, K, L, P, R, W	10	BH, FP, RF	9
Sideritis spp., Lamiaceae, IN, co	Sideritis (*S. perfoliata*); Kypriakos sideritis (*S. cypria*)	MED	108	B	67		
Thymbra capitata (L.) Cav., Lamiaceae, IN, co	Throumpi, thymari	MED, CU, LC, MP, HU	27	A, D, R, S	39	BA, BH, HS	19
Vitex agnus-castus L., Lamiaceae, IN, co	Lygaria, agnia	MED, MP	92	W, X	67		
Cinnamomum verum J.Presl, Lauraceae, --, im	Kanella	MED, CU	53	K	78	BH, FP, HS, SD	17
Laurus nobilis L., Lauraceae, IN, co/cu	Dafni	MED, CU, Co, AR, HU, PP	5	A, N, R, S, T, W	17	FP, HS	8
Punica granatum L., Lythraceae, --, cu	Rodia, rothkia	MED, MP	61	D, P, S	49		
Malva sylvestris L., Malvaceae, IN, co	Molocha, althea	MED, CU	56	A, D, R, S	34	CF	63
Ficus carica L., Moraceae, IN, co/cu	Sytzia, sykia	MED, CU	31	A, D, R, S	23	DF, FP, HS, SD	17
Morus alba L., Moraceae, --, co/cu	Sykamia, sykaminia	LC	85				
Eucalyptus spp., Myrtaceae, --, co	Efkalyptos	MED, Co	34	A, R, W	22		
Myrtus communis L., Myrtaceae, IN, co	Mersinia, mersini	MED, CU, LC, Co, RT, FH	13	A, R, S, W	11	RF	51
Syzygium aromaticum (L.) Merr. & L.M.Perry, Myrtaceae, --, im	Moschokarfi, garyfallo	MED, CU	61	D, K	59	FP, SD	41

Olea europaea L., Oleaceae, IN, cu	Elia, agrielia	MED, CU, LC, Co, RT	10	A, D, F, H, K, P, R, S	3	C, CF	48
Oxalis pes-caprae L., Oxalidaceae, --, co	Xinoudi	CU	122			RF	71
Papaver rhoeas subsp. *rhoeas*, Papaveraceae, IN, co	Paparouna, peteinos, kopela	CU, RT, MP	75			CF	71
Papaver somniferum L., Papaveraceae, --, co	Chaskasin	MED	95	P	57		
Sesamum indicum L., Pedaliaceae, --, cu	Sisami	CU	64			NCF, HS, SD	21
Cedrus brevifolia (Hook. f.) A. Henry, Pinaceae, EN, co	Kedro	MED	122	R	78		
Pinus spp., Pinaceae, IN, co	Pefkos	MED, CU, MP	43	R, S	45	SD	71
Piper nigrum L., Piperaceae, , im	Artymata, piperi, mavro piperi	MED, CU	53	A	67	FP	42
Plantago coronopus L., Plantaginaceae, IN, co	Lithospastos, lithospasto	MED	73	U	47		
Plantago lanceolata L., Plantaginaceae, IN, co	--	MED	119	R, S	67		
Plantago major L., Plantaginaceae, IN, co	Pentanevro, lithospastos	MED, CU	58	S, U, W	35	NCF	71
Platanus orientalis L., Platanaceae, IN, co	Platanos	MED	122	S	78		
Cynodon dactylon (L.) Pers., Poaceae, IN, co	Arkasto. erkastos, lithospasto	MED	105	U	63		
Oryza sativa L., Poaceae, --, im	Ryzi	MED	122	D	78		
Triticum turgidum subsp. *durum* (Desf.) Husn., Poaceae, --, cu	Sitari	CU	85			CF, FP, SD	36
Zea mays L., Poaceae, --, cu	Sitaropoula	MED	119	U	76		
Portulaca oleracea L., Portulacaceae, IN, co	Glystirida, antrakla	MED	122	T	78		
Nigella sativa L., Ranunculaceae, --, cu	Mavrokokkos	CU	122			HS	71
Rhamnus alaternus L., Rhamnaceae, IN, co	Chrysoxylia	CU, MP	92			BA	71
Agrimonia eupatoria L., Rosaceae, IN, co	Agrimonio, fonochorto	MED	122	R	78		
Crataegus spp., Rosaceae, IN, co/cu	Mosfilia (*C. azarolus*), kotzinomosfilia (*C. monogyna*), krataigos	MED, CU, CC	18	A, D, K, N, T, Y	13	BH, RF, SD	13
Cydonia oblonga Mill., Rosaceae, --, cu	Kydonia	MED, CU	29	D, N	30	BA, DF, SD	11
Malus domestica Borkh., Rosaceae, --, cu	Milia	MED, CU	43	A, R, S, T	52	BA, DF, SD	15
Mespilus germanica L., Rosaceae, --, cu	Pomilithkia	CU	108			RF	64
Prunus armeniaca L., Rosaceae, --, cu	Chrysomilia	CU	122			SD	71
Prunus avium L., Rosaceae, --, cu	Kerasia	MED, CU, MP,	17	A, D, K, R, U, X, Y	5	BA, BC, BH, RF, SD	14
Prunus cerasus L., Rosaceae, --, cu	Vyssinia	MED	122		105		
Prunus domestica L., Rosaceae, --, cu	Damaskinia	MED, CU	73	D	55	BA	71
Prunus domestica subsp. *syriaca*, Rosaceae, --, cu	Marapellia	CU	95			RF, SD	48
Prunus dulcis (Mill.) D. A. Webb, Rosaceae, --, cu	Amygdalia, athasia	MED, CU, LC, Co	21	A, D, R, S, W	18	BA, CF, SD	26
Prunus mahaleb L., Rosaceae, --, im	Kerasia, agriokerasia	CU	108			HS	64
Prunus persica (L.) Batsch, Rosaceae, --, cu	Rodakinia	MED, CU	67	D,	78	DF, RF, SD	39
Pyrus communis L., Rosaceae, --, cu	Achladia, appidkia	MED, CU	72	D	78	SD	55

Rosa damascena Mill., Rosaceae, --, cu	Triantafyllia i damaskini, triantafyllia tou Agrou	MED, CU, LC, Co, RT	2	D, F, L, P, S, W	4	HS, SD	4
Rosa spp., Rosaceae, IN, co	Arkotriantafyllia, agria triantafyllia, atzoulovatos, kynorodo, pemperissia, rodi tou Troodous (*R. micrantha* subsp. *Chionistrae*), mousietia (*R. canina*)	MED, CU, Co, CC	25	A, D, L, R, S, T, U, Y	16	BA, BH, RF, SD	36
Rubus sanctus Schreb., Rosaceae, IN, co	Vatos, imeros vatos, vramos, vatomouria, mouria	MED, CU	37	A, B, D, F, T	26	BH, RF	32
Cinchona officinalis L., Rubiaceae, --, im	Kinino	MED	122	A	78		
Citrus × aurantium L., Rutaceae, --, cu	kitromilia	CU, Co	51			C, FP, SD	24
Citrus × sinensis (L.) Osbeck, Rutaceae, --, cu	Portokalia	MED, CU	99		105	SD	71
Citrus limon (L.) Osbeck, Rutaceae, --, cu	Lemonia	MED, CU, Co	46	D, K, P, R	37	C, FP	44
Ruta chalepensis L., Rutaceae, IN, co	Apiganos	MED, AR	108		105		
Aesculus hippocastanum L., Sapindaceae, --, cu	Agriokastania	MED	122		105		
Verbascum sinuatum L., Scrophulariaceae, IN, co	Flomos	MED, FH	85	H, R	63		
Capsicum annuum L., Solanaceae, --, cu	Piperia	MED	119	S, W	67		
Capsicum frutescens L., Solanaceae, --, cu	Kokkino kaftero piperi, kafteri piperia, piperouthkia apsera	MED, CU, LC	42	A, S	46	FP, HS	40
Lycopersicon esculentum Mill., Solanaceae, --, cu	Tomata, ntomata	MED, CU	69	S	57	CF	64
Solanum tuberosum L., Solanaceae, --, cu	Patata	MED, LC	61	A, S	43		
Styrax officinalis L., Styracaceae, IN, co	Steratzia, tsounna	CU, Co, RT, FH	45			SD	71
Urtica spp., Urticaceae, IN, co	Tsouknida, tsouknitha, tsiknitta	MED, CU, LC, CC	19	B, D, K, L, R, T, U, W	14	BH, CF, NCF, HS	20
Aloysia citrodora Palau, Verbenaceae, --, cu	Alouiza	MED	122	T	78		
Viola odorata L., Violaceae, IN, co	Violetta	CU	122			BA	71
Vitis vinifera L., Vitaceae, --, cu	Ampeli	MED, CU, LC, Co, RT, HU, CC	1	A, D, F, K, L, N, R, S, W	1	BA, BH, C, DF, CF, FP, RF, SD	1
Aloe vera (L.) Burm. f., Xanthorrhoeaceae, --, cu	Aloi	MED	105	S	63		
Asphodelus ramosus L., Xanthorrhoeaceae, IN, co	Spouthoulla	MED	78	H, S	43		
Tribulus terrestris L., Zygophyllaceae, IN, co	Trivoli	MED, LC	99	U	78		

*Use categories: Medicinal (MED), Culinary (CU), Livestock care (LC), cosmetics (CO), manufacture-processing (MP), religious traditions (RT), animal repellent (AR), crop care (CC), household use (HU), fishing-hunting (FH), trade (TR), poisonous plants (PP). Medicinal sub-categories (according to ICPC-2): Skin (S), digestive (D), general & unspecified (A), respiratory (R), eye (F), cardiovascular (K), urological (U), psychological (P), musculoskeletal (L), endocrine/metabolic & nutritional (T), pregnancy, childbearing (W), neurological (N), blood, blood forming organs and immune mechanism (B), female genital (x), male genital (Y), ear (H). Culinary sub-categories: Sweets & desserts (SD), food preservation (FP), herbs & spices, (HS), hot beverage (tea; BH), cooked food ingredient (CF), raw food (RF), alcoholic beverage (BA), non-cooked food ingredient (NCF), dried fruits (DF), condiment (other; C), pickled food (PF), cold beverage (BC), smoke flavoring (SF).

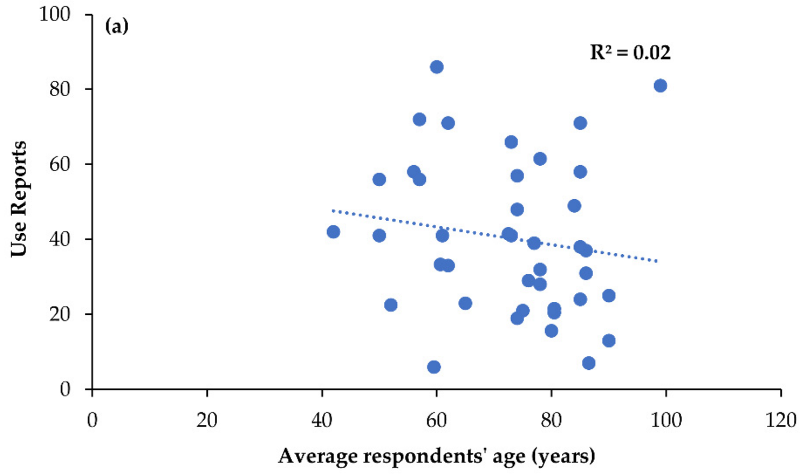

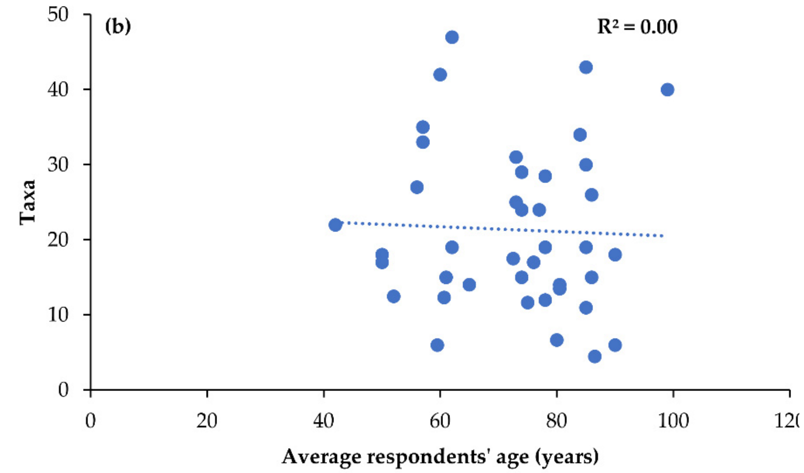

Figure S2. (a) The relationship between the average use reports (i.e. use reports mentioned by a group divided by the number of respondents) mentioned by each group of respondents and the average age of the respondents within a group. (b) The relationship between the average number of taxa (i.e. number of taxa mentioned by a group divided by the number of respondents) mentioned by each group of respondents and the average age of the respondents within the group.
